# Assessing Cardiovascular Target Attainment in Type 2 Diabetes Mellitus Patients in Tertiary Diabetes Center in Romania

**DOI:** 10.3390/ph17091249

**Published:** 2024-09-23

**Authors:** Teodor Salmen, Valeria-Anca Pietrosel, Delia Reurean-Pintilei, Mihaela Adela Iancu, Radu Cristian Cimpeanu, Ioana-Cristina Bica, Roxana-Ioana Dumitriu-Stan, Claudia-Gabriela Potcovaru, Bianca-Margareta Salmen, Camelia-Cristina Diaconu, Sanda Maria Cretoiu, Anca Pantea Stoian

**Affiliations:** 1 Doctoral School, “Carol Davila” University of Medicine and Pharmacy, 050474 Bucharest, Romania; teodor.salmen@drd.umfcd.ro (T.S.);; 2 DiabetMed Clinic, 052034 Bucharest, Romania; 3 Department of Medical-Surgical and Complementary Sciences, Faculty of Medicine and Biological Sciences, “Ștefan cel Mare” University, 720229 Suceava, Romania; delia.pintilei@usm.ro; 4 Department of Diabetes, Nutrition and Metabolic Diseases, Consulted Medical Centre, 700544 Iasi, Romania; 5 Department of Internal, Family and Occupational Medicine, “Carol Davila” University of Medicine and Pharmacy, 020021 Bucharest, Romania; 6 Doctoral School, University of Medicine and Pharmacy, 200349 Craiova, Romania; 7 5th Department, “Carol Davila” University of Medicine and Pharmacy, 050474 Bucharest, Romania; camelia.diaconu@umfcd.ro; 8 Department of Morphological Sciences, Cell and Molecular Biology and Histology, “Carol Davila” University of Medicine and Pharmacy, 050474 Bucharest, Romania; sanda.cretoiu@umfcd.ro; 9 Diabetes, Nutrition and Metabolic Diseases Department, “Carol Davila” University of Medicine and Pharmacy, 050474 Bucharest, Romania; anca.stoian@umfcd.ro

**Keywords:** cardiovascular disease, type 2 diabetes mellitus, LDL-cholesterol, risk, cardio-renal protection, statin, glucagon-like peptide-1 receptor agonists, sodium-glucose co-transporter-2 inhibitors

## Abstract

Introduction: Type 2 diabetes mellitus (T2DM) and cardiovascular disease (CVD) share a bidirectional link, and the innovative antidiabetic molecules GLP-1 Ras and SGLT-2is have proven cardiac and renal benefits, respectively. This study aimed to evaluate CV risk categories, along with lipid-lowering and antidiabetic treatments, in patients with T2DM from a real-life setting in Romania. Material and Methods: A cross-sectional evaluation was conducted on 405 consecutively admitted patients with T2DM in an ambulatory setting, assessing them according to the 2019 ESC/EAS guidelines for moderate, high, and very high CV risk categories. Results: The average age of the group was 58 ± 9.96 years, with 38.5% being female. The mean HbA1C level was 7.2 ± 1.7%. Comorbidities included HBP in 88.1% of patients, with a mean SBP and DBP of 133.2 ± 13.7 mm Hg and 79.9 ± 9 mm Hg, respectively, and obesity in 66.41%, with a mean BMI of 33 ± 6.33 kg/m^2^. The mean LDL-C levels varied by CV risk category: 90.1 ± 34.22 mg/dL in very high risk, 98.63 ± 33.26 mg/dL in high risk, and 105 ± 37.1 mg/dL in moderate risk. Prescribed treatments included metformin (100%), statins (77.5%), GLP-1 Ras (29.4%), and SGLT-2is (29.4%). Conclusions: In Romania, patients with T2DM often achieve glycemic control targets but fail to meet composite targets that include glycemic, BP, and lipid control. Additionally, few patients benefit from innovative glucose-lowering therapies with proven cardio-renal benefits or from statins.

## 1. Introduction

The incidence of Type 2 Diabetes Mellitus (T2DM) is rising steadily, surpassing the predicted prevalence rates and becoming one of the most significant pandemics of this century. Additionally, this condition is a leading cause of death, disability, and lost years of life, posing a substantial burden to healthcare systems [1,2,3]. The PREDATORR study conducted in Romania in 2016 reported an undiagnosed DM prevalence of 2.4% among the population and a diagnosed prevalence of 11.6% among individuals aged 20 to 79 years [4].

The lack of proper glycemic and metabolic control can result in the development and progression of complications associated with T2DM, with high mortality rates. To mitigate these risks, the American and European Diabetes Associations have issued guidelines recommending the avoidance of therapeutic inertia and the use of innovative antidiabetic agents with cardioprotective and renal protective effects, such as GLP-1 Ras and SGLT-2is [2,5,6,7]. The efficacy of these agents was demonstrated through large-scale CVOTs, despite the heterogeneity of the various agents within the two pharmacological classes. These trials demonstrated improved metabolic control, reflected by a better management of weight, glycemic levels, BP, and lipid profiles. Additionally, they provided CV protection, as indicated by a reduction in major CVEs, including stroke and myocardial infarction, as well as a decreased risk of HF hospitalization or CV mortality [8,9].

The most widely prescribed medication for the treatment of T2DM globally is metformin, a biguanide derivative known as dimethylbiguanide. It is commonly prescribed as a first-line therapy, either alone or in combination with other medications. However, current guidelines recommend that innovative medication classes, such as GLP-1 Ras and SGLT-2is, should be used in T2DM patients regardless of their glycemic control, with this approach aiming to reduce CV and renal risks. The CV effects of metformin are limited, but it is known to reduce oxidative stress and inflammation and improve endothelial function [10]. There are also some nutritional supplements that have been shown to reduce oxidative stress [11]. Additionally, physical activity can help reduce oxidative stress and contribute to weight loss [12]. Given these factors, it is nevertheless still important to focus on patient education and lifestyle advice for those with T2DM and other CV risk factors. Education should include guidance on physical activity, understanding the impact of carbohydrates on blood glucose levels, and overall dietary management. DM can impact patients’ functionality and lead to disability, often due to complications like diabetic neuropathy or dietary restrictions. These issues can be addressed through effective assessments, education, and comprehensive rehabilitation programs [13].

CVD includes ischemic coronary disease, stroke, and peripheral arterial disease, and constitutes the leading cause of mortality globally, greatly impairing individuals’ quality of life [1,2,14]. The most frequent manifestation of CVD, CHD, was reported in 2019 with 197 million cases of incidence and 9.14 million fatalities by the Global Burden of Disease Study [15]. The 2022 update of the AHA reports a 7.2% CHD prevalence in individuals above 20 years old in the United States [16]. In the EU, in 2020, 1.70 million deaths resulted from diseases of the circulatory system, equivalent to 32.7% of all deaths as compared to 22.5% for the second most prevalent cause of death, cancer. These are alarming percentages, with more than half of all deaths registered in Central-East countries (60.6% in Bulgaria, which leads the ranking, shortly followed by Romania with 55.1% deaths) and Baltic States Lithuania and Latvia [17]. The 2019 report from the ESC Atlas stated that 15% of CVD deaths in Europe are due to poorly controlled DM [18].

CVD and T2DM are part of cardio-reno-metabolic syndrome; therefore, these entities have a bidirectional link [19]. The level of risk is further enhanced in the case of associations with additional standard risk factors, such as excess weight, high BP (HBP), dyslipidemia, smoking status, and physical inactivity, especially when they overlap with a hyperglycemic state [20]. The emergence of CVEs is more likely to occur in individuals with elevated levels of total cholesterol, particularly high levels of LDL-C. A reduction of one mmol/L in LDL-C levels can substantially decrease the risk of CVEs, regardless of the baseline level [21]. The long-term exposure to blood glucose levels, specifically the HbA1C level, is closely associated with CVEs. An increase of 1% in HbA1C is linked to a 20% increase in the risk of atherosclerotic CVEs. Conversely, maintaining the HbA1C level below 7% for over 10 years significantly decreases the risk of CVD [22,23].

To enhance CV risk stratification, various predictive models have been developed to enable a multifactorial approach to CVD. These models comprehensively identify and quantify risk factors. The early identification of high risk individuals is critical, particularly since DM frequently coexists with CVD and is a significant risk factor itself. Therefore, in trials, risk stratification has been continuously refined to include the most relevant variables for optimal estimation, supporting personalized care management, meaning targeted therapy and specific treatment goals [23].

Between 2019 and 2023, continuous efforts were made to improve CV risk stratification in patients with DM. The 2019 ESC/EAS guidelines categorized T2DM patients as very high, high, or moderate risk, eliminating the need for the SCORE assessment. In 2021, the ESC Guidelines recommended using the ADVANCE or DIAL models for CV risk assessment in DM patients. The 2023 update introduced the SCORE2-Diabetes algorithm, which utilizes factors like age, gender, smoking status, BP, cholesterol levels, and renal health to classify CV risk into low, moderate, high, or very high categories. [11,16,24,25]. These risk factors are also addressed by the American Diabetes Association (ADA) guidelines, which outline the four pillars of DM management: glucose, BP, lipid, and weight control. The guidelines recommend using glucose-lowering medications that offer cardiac or renal benefits [5,14].

Despite these advances in treatment guidelines and risk stratification models that provide clear guidance on selecting therapeutic agents according to the CV risk groups, a significant gap remains in understanding how many patients with T2DM achieve the recommended therapeutic targets and how closely their medications are prescribed according to these guidelines. This research aims to bridge this gap by offering updated insights into the management of T2DM patients as it evaluates the achievement of specific therapeutic targets—HbA1C, LDL-C, and BP—both individually and collectively. The study also examines the utilization rates of statins and novel antidiabetic therapies with established cardio-renal benefits, including GLP-1 Ras and SGLT-2is. Specifically, it assesses the proportion of patients classified according to the 2019 ESC/EAS CV risk categories and determines the percentage of patients prescribed statins and novel antidiabetic agents with proven cardio-renal protective effects. By analyzing these factors, the study aims to enhance awareness of a critical public health issue and provide guidance for future clinical decisions.

## 2. Results

The demographic characteristics of the 405 patients included a mean age of 58 ± 9.96 years, with 38.5% females, and a median duration of T2DM of 6 (0, 59) years.

Our cohort’s unmodifiable risk factors consisted of age, gender, and duration of DM, while the modifiable risk factors included poorly controlled DM, obesity, dyslipidemia, and BP. Additional patient characteristics are detailed in Table 1.

Among the patients, 66.41% were obese, with a mean body mass index of 33 ± 6.33 kg/m^2^; 88.1% had high BP, with a mean SBP of 132.3 ± 13.7 mm Hg and a mean DBP of 79.9 ± 9 mm Hg. Smoking was reported by 20% of the patients, and 34.32% had atherosclerotic CVD. The mean LDL-C values were distributed as follows: 90.1 ± 34.22 mg/dL in the very high CV risk category, 98.63 ± 33.26 mg/dL in the high CV risk category, and 105 ± 37.1 mg/dL in the moderate CV risk category. The mean HbA1C level was 7.2 ± 1.7%, and the lipid profile showed a mean total-C of 168.8 ± 42.6 mg/dL, a mean HDL-C of 44.7 ± 14.2 mg/dL, a mean LDL-C of 91.5 ± 34.1 mg/dL, and a median TG level of 156 mg/dL (ranging from 38 to 1080). 

Table 1 provides a summary of the prescribed glucose-lowering therapies by category, including metformin (100%), insulin (23.9%), GLP-1 Ras (29.4%), and SGLT-2is (29.4%). Additionally, the table details the use of other therapies targeting the CV risk factors, such as ACEi/ARBs (69.1%), statins (77.5%), beta-blockers (60.7%), calcium channel blockers (24.4%), and diuretics (37.8%).

Table 2, Table 3 and Table 4 illustrate the subsequent distribution of the 405 patients into the CV risk categories, with 340 patients (83.9%) in the very high CV risk category, 62 patients (15.3%) in the high CV risk category, and 3 patients (0.8%) in the moderate CV risk category. A concise description of our results is shown in the tables, with the number of patients who achieved their targets for LDL-C, HbA1C, and BP, both separately and in combination. They also summarize the use of innovative antidiabetic and lipid-lowering medications. According to the 2019 ESC/EAS Guidelines for LDL-C and the 2019 ADA Guidelines for HbA1C and BP, data about the patients with very high, high, and moderate CV risk are presented in Table 2, Table 3, and Table 4, respectively.

## 3. Discussion

This study provided an illustrative image of the CV risk categories for T2DM patients from a 2019 ambulatory setting in a Romanian tertiary care center. These categories were defined using the 2019 ESC/EAS guidelines for LDL-C and the 2019 ADA guidelines for HbA1C and BP, in effect at the time of the cohort’s assessment. Additionally, we evaluated the prescription rates of statins and innovative cardio-renal protective antidiabetic drugs, specifically GLP-1 Ras and SGLT-2is, across these CV risk categories.

The cohort’s mean age was 58 ± 9.9 years, highlighting that these molecules were prescribed early. With a median DM duration of 6 years, the patients’ metabolic control was borderline, evidenced by a mean HbA1C of 7.2 ± 1.7%. This data aligns with other cohort studies of T2DM patients, such as Vintila et al. [26], who reported a mean age of 71 years and mean HbA1C of 7.2%; Reurean-Pintilei et al. [23], with a mean age of 62.9 ± 7.7 years and mean HbA1C of 7.1 ± 1.3%; and Cokolic et al. [27], with a mean age of 63.5 ± 10.7 years, a mean DM duration of 8.9 ± 7.1 years, and a mean HbA1C of 7.3 ± 1.5%.

Obesity, a key risk factor for developing DM, affects a substantial portion of the population, accounting for 66.41% with an average BMI of 33 ± 6.33 kg/m^2^, similar to other Romanian reports [23] and higher than other Eastern European countries and the USA, which report a 20–30% prevalence [28,29].

HBP was recorded in an overwhelming percentage in our cohort (88.1% of patients), similar to a recent report by Reurean-Pintilei et al. [23]. The percentage is almost double than the SEPHAR III study’s reports of 45.1%, but this was expected, because in patients with T2DM, usually higher rates of HBP as compared to the general population are encountered [30,31].

When compared with reports from other standard of care approaches from similar time frames in Romania [23], Scotland [32], and Denmark [33], the included patients were younger (58 years old as compared to 62 years old, 67 years old, and 72 years old, respectively), suggesting that the risk factors appear early, especially in Eastern European populations. Moreover, the patients had a shorter DM duration (6 years versus 9 years and 7.8 years). In terms of CVEs, the 34.32% of CVEs is similar to the one reported for Scotland (32%), but bigger than other regions in Romania (13.9%) or Denmark (21.4%) report. On the other hand, the mean levels of HbA1C, SBP, and BMI are similar.

When comparing the CV risk category percentages according to the 2019 ESC/EASD guidelines with a similar Romanian study by Reurean-Pintilei et al. [23], we observed fewer patients in the very high CV risk category, 83.9% versus 92.7%, and more in the high CV risk category, 15.3% versus 1.12%, with a lower moderate CV risk category percentage of 0.8% versus 6.7%. When comparing with the Santorini study [34], which included over 9000 patients from 14 European but non-eastern countries, we observed a lower median LDL-C level of 82 mg/dL and larger percentages for the very high CV risk of 91% and the high CV risk of 6.5% as compared to our results. Because only four patients (1.18%) achieved all of the HbA1C, LDL-C, and BP targets in the very high CV risk category, it is important to emphasize that ASCVD was present in almost one of three enrolled patients, similar to the data reported by McGurnaghan et al. [33], but almost triple compared to Reurean-Pintilei et al. [23]. On the other hand, if taken individually, 49 patients (14.42%) achieved the BP target alone, 138 patients (40.59%) achieved the HbA1C target, and 45 patients (13.23%) achieved the LDL-C target. Another important aspect found in the patients that reached all of the three targets simultaneously is the low rate of prescription of innovative cardio-renal protective medication; respectively, one patient received GLP-1 Ras and two patients received statins. It can be inferred that the patients included in the study exhibited a commendable level of metabolic control, which suggests that these individuals were proactive in managing their health by consistently attending medical check-ups and maintaining a healthy lifestyle. Therefore, the low prescription rate of the innovative pharmaceuticals may be deceptively minimal, particularly given that these medications were prescribed exclusively in accordance with the stringent and inflexible criteria established by the national prescription guidelines in 2019, when the HbA1C levels exceeded 7%.

An important aspect in our study is that 13.23% patients from the very high CV risk category met the 2019 recommended guidelines for the LDL-C targets, more than double the previously reported data from a Romanian study, as well [23] as the DA VINCI study [35], where the percentages were 5% and 4%, respectively. The previous rates of achieved LDL-C targets, according to the 2016 ESC/EAS guidelines, were similar to our reported data but higher than the ones reported in the DA VINCI study, which included over 2000 Romanian patients; therefore, we can conclude that the physician’s underestimation of the patient’s risk and fear of escalating lipid-lowering treatment in groups of patients other than the very high CV risk category are aspects to be taken into account [31]. Still, in our cohort, 77.5% of patients were prescribed statins, more than the 67.8% reported in other regions of the same country [23], with both percentages being higher than the 48.4% reported by the Santorini study [34]. Delving deeper, only 9.7% of the patients in the very high CV risk category treated with statins met the LDL-C target, a figure that lies between the 15% reported by Morieri et al. [36] and the 2.7% reported by Reurean-Pintilei et al. [23]. Despite the recommendations from several medical societies, including the American Association of Clinical Endocrinologists, the ESC/EAS guidelines for dyslipidaemia management, and the American College of Cardiology/American Heart Association Task Force [15,37,38], which advocate for an LDL-C target below 55 mg/dL for those at very high or extreme CV risk, the consistently low achievement rates highlight an urgent need to increase awareness and adopt more aggressive lipid management strategies in these populations [23,26,34,35,36]. Expanding our analysis to the high and moderate CV risk categories, we find that no patients simultaneously met the LDL-C, BP, and HbA1C targets.

Statin use was lower than in other Romanian data, but with similarities with Scotland and Denmark, while ACEi/ARBs were lower in both Romanian studies as compared to the other two countries [23,32,33]. Moreover, even if in our cohort the rates of SGLT-2is and GLP-1 Ras were low, they were recommended more than in previously reported data on similar cohorts, 29.4% versus 3.9% and 29.4% versus 8.1%, respectively, and also in higher proportion for the very high and high CV risk categories. The trend of under-prescribing these innovative medications is similar to the reported data of Vencio et al. [39], where 15% of patients received SGLT-2is and 9% received GLP-1 Ras, or the Discover study by Arnold et al. [40], where almost 16.1% of patients received these types of medications at follow-up, but with variable values between the included countries. A rate over 63% was, on the contrary, reported by countries with smaller economic power from Southern and Eastern Europe, as reported by Banach et al. [41]. In Denmark and Scotland, SGLT-2is and GLP-1 Ras were also under-prescribed, with 4% and 2% and 8% and 5.4%, respectively [32,33].

An interesting fact is the high percentage of metformin prescription that was present in all patients from our cohort, in comparison with the data from Scotland, Denmark, or Romania, with rates of 57%, 54%, and 87%, respectively; for insulin, the 23.9% rate was similar to the data reported in Romania of 25%, but higher than the ones from Scotland or Denmark of, respectively, 11% and 19.5% [23,32,33].

In a broader context, simultaneously with the development of these novel molecules and the updates of the medical guidelines on their use, medical research showed more clearly that DM is evolving silently and leads to the appearance of CVEs and high mortality rates; hence, a more accurate stratification for CV risk is essential. Because of DM and CVD’s silent progression, complementary investigations such as magnetic resonance imaging and coronary computed tomography angiography are needed both in primary and secondary prevention, in order to be able to include specific therapies or interventions in the management of very high risk patients [42]. Another approach to refining CV risk involves the early assessment of atherosclerosis to detect progression towards plaque instability or rupture using biological markers. In this context, Lp(a) is recognized as both an independent and causal risk factor for ASCVD due to its inflammatory, prothrombotic, and atherogenic effects. It is a primary target of the latest lipid-lowering therapies, including proprotein convertase subtilisin/kexin type 9 inhibitors, small interfering RNA, and inhibitory antisense oligonucleotides. However, the implementation of Lp(a) screening and treatment faces significant challenges, including limited physician awareness, the absence of consensus guidelines amid ongoing research, and high costs, with cost-effectiveness data being both scarce and inconsistently assessed [15,43,44,45].

This study’s strengths include providing important insights into T2DM among European patients, particularly focusing on the underrepresented Romanian demographic, utilizing a cross-sectional design. This approach allows for a comprehensive snapshot of current disease management practices and CV risks at a specific point in time, facilitating an immediate comparison with other countries. The utilization of the 2019 ESC/EAS and ADA guidelines in use at the time of the evaluation enhances the depiction of the CV risk categories, while the detailed data collection underscores significant areas of underutilization due to factors such as physician unawareness and medical inertia. Moreover, the comparative analysis not only underscores the regional variations that may influence disease management outcomes, but also identifies potential areas for improvement in local healthcare strategies. This is essential for acknowledging disparities compared to other regions or populations, particularly given the underrepresentation of Romanian patients in studies that often focus on Western European populations. The fact that our cohort consisted of younger patients can be also considered a strength, as it adds important data on the real-life clinical status of the working-age population with DM.

The cross-sectional design of our study serves as both a strength and a limitation. So, the study’s main limitations are the cross-sectional design, the exclusively Romanian cohort from a single center, the relatively small sample, and the lack of data about physician inertia levels and cost perceived barriers in prescribing the innovative molecules. This design limits our ability to infer causality or track changes over time, preventing an evaluation of T2DM progression, the evolution of prescription patterns, and the long-term effectiveness of treatment strategies. Additionally, with a focus predominantly on a Romanian cohort, our study may have a limitation on the generalizability of the findings to other regions or populations. The specific healthcare setting, different cultural factors, and economic conditions in different geographical regions of Romania may not be representative of other European or global contexts. This study included participants from a single tertiary care center, leading to a potential selection bias, as we are unable to accurately reflect the broader population of T2DM patients in Romania or elsewhere. This aspect could influence the observed prevalence of CV risk factors and the reported prescription rates of medication. Lastly, while providing data on prescription patterns (although not including data on dipeptidyl-peptidase 4 inhibitors or sulphonylurea) and risk categorization, our study does not offer the explanations behind physician inertia or the barriers to the adoption of innovative treatments beyond cost and awareness. It should be noted that in 2019, prescription practices were subject to restrictions under the Romanian health insurance system.

In order to mitigate the issues identified in our study and enhance T2DM management, several measures could be recommended as possible perspectives. This should start with future longitudinal studies to aid in policy adjustments and to improve educational and policy interventions. A more detailed exploration of these factors could offer clearer guidance for interventions designed to enhance T2DM management. Simplifying the national prescribing criteria would facilitate easier access to innovative antidiabetic drugs for high risk patients. Targeted educational programs could increase healthcare providers’ awareness of the latest guidelines and emphasize comprehensive treatment strategies. Establishing regular audits of prescription patterns, coupled with feedback mechanisms, would potentially encourage guideline adherence and improve prescribing practices. Additionally, patient engagement and education would empower individuals to actively participate in their treatment, thereby enhancing adherence and outcomes.

## 4. Materials and Methods

### 4.1. Study Design and Patients

This single-center, consecutive-case investigation was cross-sectional and population-based, directed as part of a sub-analysis derived from a retrospective investigation. The principles outlined in the Declaration of Helsinki were followed and protocol number 5591, dated 17 November 2022 from the Institutional Ethics Committee of the N Paulescu National Institute for Diabetes Mellitus, Nutrition, and Metabolic Disorders in Bucharest, Romania, was obtained. Initially, between January and July in 2019, 477 patients with T2DM who met the inclusion criteria were sequentially invited to participate during their routine visits, during which their medical records were gathered. Subsequently, data analysis was performed on the records of the 405 patients who provided informed consent. The following criteria were used to determine eligibility for the study: adults over the age of 18, the provision of informed consent, a confirmed diagnosis of T2DM for at least six months, and treatment with the standard of care maximum tolerated doses for the associated conditions for at least six months prior to the study. The study also excluded patients who were under 18 years of age, those who were diagnosed with other types of DM than T2DM (such as Type 1 DM or secondary DM), those who did not provide signed informed consent, and those who had severe or acute heart failure, renal insufficiency, or hepatic insufficiency.

The patient data were collected from hospital reports and included demographics (age, gender, and residential background), anthropometrics (height, weight, and BMI), concurrent conditions (e.g., smoking status, HBP, dyslipidemia, and atherosclerotic CV disease), laboratory test results (HbA1C, LDL-C, total-C, high-density lipoprotein cholesterol (HDL-C), triglycerides, uric acid, UACR, and eGFR), and treatment specifics (such as antidiabetics, BP, and lipid-lowering medications).

### 4.2. Statistical Analysis

The categorical data were expressed as numbers and percentages. Continuous variables were evaluated using the Kolmogorov–Smirnov test, and depending on their distribution, were expressed as either the median (IQR) or the mean ± SD. We divided the population data into three different groups according to the antidiabetic treatments (metformin, metformin + SGLT-2is, and metformin + GLP-1 Ras, respectively). This information was organized into Excel spreadsheets and analyzed using Excel 2019 software.

### 4.3. Patients’ Stratification

The patients were stratified into CV risk categories following the 2019 ESC/EAS guidelines, namely the moderate, high, and very high CV risk groups.

The low risk category was not included due to the presence of T2DM in all patients, which represented that they were in the most favorable scenario in the moderate risk category. The moderate CV risk category included the patients with T2DM younger than 50 years of age, with a DM duration of less than 10 years, and without additional risk factors. The high risk category encompassed the patients with T2DM without TOD, with a DM duration of at least ten years, or presenting an additional risk factor.

The very high risk category included the patients with T2DM and TOD, T2DM and at least three major risk factors, or documented ASCVD. Documented ASCVD included a history of ACS, stable angina, coronary revascularization procedures (PCI, CABG, and other arterial revascularization), stroke, TIA, or peripheral arterial disease. Unequivocal documentation on imaging involved findings predictive of clinical events, such as significant plaque on a coronary angiography or CT scan or notable carotid ultrasound results.

The goals for each CV risk category were established based on the 2019 ESC/EAS standards for LDL-C and the 2019 ADA guidelines for HbA1C and BP:

i.   Moderate CV risk category: LDL-C < 100 mg/dL, HbA1C < 7%, and BP < 130/80 mmHg.
ii.  High CV risk category: LDL-C < 70 mg/dL, HbA1C < 7%, and BP < 130/80 mmHg.
iii. Very high CV risk category: LDL-C < 55 mg/dL, HbA1C < 7%, and BP < 130/80 mmHg.

Upon assessing the biological and paraclinical parameters in accordance with the guidelines, we evaluated the use of statins and novel T2DM cardio-renal protective therapies, specifically the GLP-1 Ras and SGLT-2is classes, which were accessible in Romania in 2019.

## 5. Conclusions

Romanian patients typically fall into very high or high CV risk categories. Despite recent scientific advances, the strict criteria of the national prescribing protocols and prevalent medical inertia mean that in real-world settings, these patients rarely achieve the optimal prescriptions of lipid-lowering and innovative antidiabetic drugs, such as SGLT-2is and GLP-1 Ras. Conversely, they often meet BP or glycemic targets individually. Given that patients with T2DM frequently exhibit additional CV risk factors, improving their prognosis requires a comprehensive treatment evaluation that simultaneously targets BP, HbA1C, and LDL-C. The failure to meet the guideline recommendations for cardio-renal benefits is a critical issue, particularly for those with T2DM and high CV risk. A longitudinal follow-up of this cohort will determine whether there are improvements in guideline adherence, better target achievement, and enhanced overall care and potential guidance.

## Abbreviation List

ACEi/ARBsAngiotensin-Converting Enzyme Inhibitors/Angiotensin Receptor Blockers ACSAcute Coronary SyndromeAHAAmerican Heart AssociationASCVDAtherosclerotic Cardiovascular DiseaseBMIBody Mass IndexBPBlood PressureCHDCoronary Heart DiseaseCVCardiovascularCVDCardiovascular DiseaseCVEsCardiovascular EventsCVOTsCardiovascular Outcome TrialsDBPDiastolic Blood PressureDMDiabetes MellitusEASEuropean Atherosclerosis SocietyESCEuropean Society of CardiologyEUEuropean UnionGLP-1 RasGlucagon-Like Peptide-1 Receptor AgonistsHbA1CA1c HaemoglobinHBPHigh Blood PressureHDL-CHigh-Density Lipoprotein CholesterolHFHeart FailureIQRInterquartile RangeLDL-CLow-Density Lipoprotein CholesterolLp(a)Lipoprotein(a)SBPSystolic Blood PressureSDStandard DeviationSGLT-2isSodium-Glucose Co-Transporter-2 InhibitorsT2DMType 2 Diabetes MellitusTGsTriglyceridesTIATransient Ischemic AttackTODTarget Organ DamageTotal-CTotal Cholesterol

## Figures and Tables

**Table 1:** Patients’ characteristics.

Characteristic	*n* = 405
Demographics	
Age (years), mean (SD)	58 ± 9.96
Women, %, (*n*)	38.5% (156)
DM mean duration, median (25–75% IQR)	6 (2, 12)
Risk factors	
BMI (kg/m^2^), mean (SD)	33 ± 6.33
Obesity, %, (*n*)	66.41% (269)
SBP (mm Hg), mean (SD)	132.3 ± 13.7
DBP (mm Hg), mean (SD)	79.9 ± 9
HBP, %, (*n*)	88.1% (357)
Smoking status, %, (*n*)	20% (81)
HbA1C (%), mean (SD)	7.2 ± 1.7
Total-C (mg/dL), mean (SD)	168.8 ± 42.6
HDL-C (mg/dL), mean (SD)	44.7 ± 14.2
TGs (mg/dL), median (25–75% IQR)	156 (106, 206)
LDL-C (mg/dL), mean (SD)	91.5 ± 34.1
Atherosclerotic CVD, %, (*n*)	34.32% (139)
eGFR (mL/min/1.73 m^2^)	97.6 ± 16.8
LDL-C in very high CV risk category, (mg/dL), mean (SD)	90.1 ± 34.22
LDL-C in high CV risk category, (mg/dL), mean (SD)	98.63 ± 33.26
LDL-C in moderate CV risk category, (mg/dL), mean (SD)	105 ± 37.1
Glucose-lowering medication usage	
Insulin, %, (*n*)	23.9% (97)
Metformin, %, (*n*)	100% (405)
GLP-1 Ras, %, (*n*)	29.4% (119)
SGLT-2is, %, (*n*)	29.4% (119)
Other therapies	
ACEi/ARBs, %, (*n*)	69.1% (280)
Statins, %, (*n*)	77.5% (314)
Beta-blockers, %, (*n*)	60.7% (246)
Calcium channel blockers, %, (*n*)	24.4% (99)
Diuretics, %, (*n*)	37.8% (153)

**Table 2:** Very high CV risk category.

Treatment Target for Patients with Very High CV Risk Category (*n* = 340)	Patients Achieving Target	Patients with SGLT-2is Prescription	Patients with GLP-1 Ras Prescription	Patients with Statin Prescription
LDL-C < 55 mg/dL, %, (*n*)	13.23% (45)	5% (17)	3.53% (12)	9.7% (33)
HbA1C < 7%, %, (*n*)	40.59% (138)	5% (17)	19.7% (67)	27.65% (94)
BP < 130/80 mmHg, %, (*n*)	14.42% (49)	4.71% (16)	5.3% (18)	10.9% (37)
LDL-C < 55 mg/dL + HbA1C < 7%, %, (*n*)	5.88% (20)	0.59% (2)	2.06% (7)	3.53% (12)
HbA1C < 7% + BP < 130/80 mmHg, %, (*n*)	6.76% (23)	0.88% (3)	3.53% (12)	4.18% (14)
LDL-C < 55 mg/dL + BP < 130/80 mmHg, %, (*n*)	2.06% (7)	0.3% (1)	0.88% (3)	1.47% (5)
LDL-C < 55 mg/dL + HbA1C < 7% + BP < 130/80 mmHg, %, (*n*)	1.18% (4)	0	0.3% (1)	0.59% (2)

**Table 3:** High CV risk category.

Treatment Target for Patients with High CV Risk Category (*n* = 62)	Patients Achieving Target	Patients with SGLT-2is Prescription	Patients with GLP-1 Ras Prescription	Patients with Statin Prescription
LDL-C < 70 mg/dL, %, (*n*)	20.96% (13)	6.45% (4)	6.45% (4)	14.51% (9)
HbA1C < 7%, %, (*n*)	38.7% (24)	6.45% (4)	14.51% (9)	27.42% (17)
BP < 130/80 mmHg, %, (*n*)	16.13% (10)	8.06% (5)	3.23% (2)	11.29% (7)
LDL-C < 70 mg/dL + HbA1C < 7%, %, (*n*)	9.68% (6)	1.61% (1)	4.84% (3)	4.84% (3)
HbA1C < 7% + BP <130/80 mmHg, %, (*n*)	4.84% (3)	0	3.23% (2)	4.84% (3)
LDL-C < 70 mg/dL + BP < 130/80 mmHg, %, (*n*)	1.61% (1)	1.61% (1)	0	1.61% (1)
LDL-C < 70 mg/dL + HbA1C < 7% + BP < 130/80 mmHg, %, (*n*)	0	0	0	0

**Table 4:** Moderate CV risk category.

Treatment Target for Patients with Moderate CV Risk Category (*n* = 3)	Patients Achieving Target	Patients with SGLT-2is Prescription	Patients with GLP-1 Ras Prescription	Patients with Statin Prescription
LDL-C < 100 mg/dL, %, (*n*)	66.6% (2)	0	0	0
HbA1C < 7%, %, (*n*)	0	0	0	0
BP < 130/80 mmHg, %, (*n*)	33.3% (1)	0	0	0
LDL-C < 100 mg/dL + HbA1C < 7%, %, (*n*)	0	0	0	0
HbA1C < 7% + BP < 130/80 mmHg, %, (*n*)	0	0	0	0
LDL-C < 100 mg/dL + BP < 130/80 mmHg, %, (*n*)	33.3% (1)	0	0	0
LDL-C < 100 mg/dL + HbA1C < 7% + BP < 130/80 mmHg, %, (*n*)	0	0	0	0

## Data Availability

The data supporting this study’s findings are available upon request from the authors. Please note that the data are also being utilized in an ongoing doctoral project evaluating the novel antidiabetic drugs.
